# Weekly Semaglutide vs. Liraglutide Efficacy Profile: A Network Meta-Analysis

**DOI:** 10.3390/healthcare9091125

**Published:** 2021-08-30

**Authors:** Hassan A. Alsugair, Ibrahim F. Alshugair, Turki J. Alharbi, Abdulaziz M. Bin Rsheed, Ayla M. Tourkmani, Wedad Al-Madani

**Affiliations:** 1 Family Medicine Department, Prince Sultan Military Medical City, Riyadh 12624, Saudi Arabia; abdulaziz_binrsheed@yahoo.com (A.M.B.R.); aylatourkmani@gmail.com (A.M.T.); 2 Orthopedic Surgery Department, King Saud Medical City, Riyadh 12746, Saudi Arabia; ishugair@gmail.com; 3 General Authority of Statistics, Riyadh 11481, Saudi Arabia; madaniwh@gmail.com

**Keywords:** network meta-analysis, diabetes mellitus, glycemic control, HbA1c, weight, semaglutide, liraglutide, Glucagon-like peptide, GLP-1, GLP-1 RA

## Abstract

Introduction: Glucagon-like peptide 1 receptor agonist (GLP-1 RA) is a class of hypoglycemic medications. Semaglutide once-weekly (QW) and liraglutide once-daily (OD) significantly improved glycemic control compared to placebo. To date, no long-term phase III trials directly comparing semaglutide and liraglutide are available. This network meta-analysis (NMA) aims to compare the long-term efficacy of semaglutide and liraglutide. Methods: PubMed, Embase, and Cochrane Library were searched from inception until June 2019 to identify relevant articles. Nine long-term randomized controlled trials comparing once-weekly semaglutide or liraglutide with placebo or other active comparisons were identified. The outcomes of interest were changes in HbA1c and weight after 52 weeks. A Bayesian framework and NMA were used for data synthesis. This is a sub-study of the protocol registered in PROSPERO (number CRD42018091598). Results: The data showed significant superiority in HbA1c reduction of semaglutide 1 mg QW over liraglutide 1.2 and 1.8 mg with a treatment difference of 0.47% and 0.3%, respectively. Semaglutide 0.5 mg QW was found to be significantly superior to liraglutide 1.2 mg in HbA1c reduction with a treatment difference of 0.17%. Regarding weight reduction analysis, semaglutide 0.5 and 1 mg QW were significantly associated with a greater reduction than liraglutide 0.6 mg with a treatment difference of 2.42 and 3.06 kg, respectively. However, no significant reduction was found in comparison to liraglutide 1.2 and 1.8 mg. Conclusions: Semaglutide improved the control of blood glucose and body weight. The capacity of long-term glycemic control and body weight control of semaglutide appears to be more effective than other GLP-1 RAs, including liraglutide. However, considering the number of included studies and potential limitations, more large-scale, head-to-head, well-designed randomized-controlled trials (RCTs) are needed to confirm these findings.

## 1. Introduction

Type 2 diabetes mellitus (T2DM) is a progressive and complex metabolic disorder characterized by chronic hyperglycemia due to insulin resistance and pancreatic beta cell dysfunction [1]. Chronic hyperglycemia is associated with multiple complications, including retinopathy, nephropathy, and neuropathy, in addition to various manifestations of atherosclerotic cardiovascular disease (CVD) [1,2]. According to one large randomized controlled trial (RCT) involving 11,140 participants, with every 1% increase in glycosylated hemoglobin (HbA1c), risk was increased up to 40% of all-cause and cardiovascular mortality [3]. Glycemic control becomes progressively difficult with time, and advancements in therapy are needed to maintain glycemic targets [4,5].

Glucagon-like peptide 1 receptor agonist (GLP-1 RA) is a class of hypoglycemic medications that has shown benefit in glucose metabolism, beta-cell function enhancement, and weight loss promotion with a low risk of hypoglycemia [6]. GLP-1 RAs are classified into short- and long-acting preparations based on their mode of action [7,8]. Compared with other hypoglycemic medications, RCTs have reported promising long-term effects, especially with respect to cardiovascular outcomes [6]. The U.S. Food and Drug Administration (FDA) has approved several GLP-1 RAs, including exenatide twice-daily (Bid), lixisenatide once-daily (OD), liraglutide OD, exenatide once-weekly (QW), albiglutide QW, dulaglutide QW, and semaglutide QW [9,10,11,12,13].

The US FDA approved liraglutide in 2010 as a daily subcutaneous injection with therapeutic doses of 1.2 mg and 1.8 mg for T2DM [6,10,14]. In head-to-head RCTs, liraglutide showed a greater reduction in mean HbA1c and fasting plasma glucose (FPG) than exenatide Bid, exenatide QW, albiglutide QW and lixisenatide and was non-inferior to dulaglutide QW [15,16]. Additionally, RCTs reported greater weight reduction from baseline in liraglutide compared to lixisenatide, exenatide QW, dulaglutide QW, and albiglutide QW [16]. RCTs showed similar weight reduction in exenatide Bid compared to liraglutide [16]. The U.S. FDA approved semaglutide in 2017 as a once-weekly subcutaneous injection [9]. A 30-week phase III RCT on semaglutide reported a significant reduction in HbA1c and weight from baseline compared with placebo [17]. In a head-to-head comparison with dulaglutide and exenatide QW, semaglutide was superior in achieving glycemic control and weight reduction [18,19]. A review published by Courtney et al. on GLP-1 medications showed clinically significant glycemic control and weight reduction of liraglutide [20]. In two recent network meta-analyses on GLP-1 RA RCTs, semaglutide was found superior to liraglutide in glycemic control and weight reduction. However, conclusions were drawn from only data gathered at 24 ± 4 weeks [21,22].

To date, no long-term phase III trials directly comparing semaglutide QW and liraglutide OD are available. In the absence of long-term head-to-head RCTs, network meta-analysis is a statistical method that allows the estimation of the comparative effectiveness of multiple treatments [23,24]. This network meta-analysis (NMA) compared the long-term efficacy in HbA1c reduction and weight change between semaglutide QW and liraglutide OD.

## 2. Materials and Methods

This is a sub-study of the protocol registered in PROSPERO (number CRD42018091598). This network meta-analysis was conducted in accordance with the Preferred Reporting Items for Systematic Reviews and Meta-Analyses (PRISMA) statement [25].

Database Search: Electronic database search included the PubMed, Embase, and Cochrane’s Library from inception to June 2019. The key term “Liraglutide OR NN2211 OR Semaglutide OR NN9535” was used for all of the databases. The search was restricted to English, French, and Spanish language publications. The earliest publication found using the above search key-term dated back to 2001. However, early publications did not meet the inclusion criteria for this NMA. 

Study Selection: This review included double-blind, single-blind, or open-label RCTs with available data on HbA1c or weight. In which once-weekly semaglutide (0.5 mg or 1.0 mg) and liraglutide (1.2 mg or 1.8 mg) compared with other active intervention or placebo. RCTs with adults aged at least 18 years with T2DM and a duration of ≥52 weeks on intervention were only included. Final end-point data were used for the analysis of RCTs longer than 52 weeks. Nonrandomized, experimental studies, crossover trials, and reviews in addition to studies with less than fifty participants were excluded. The eligibility of included studies was assessed independently by two reviewers (A.H.A. and A.I.F.). Discrepancies were resolved by a third reviewer (A.T.J.).

Data Extraction and Quality Evaluation: Unified extraction forms were used to extract the following data: (1) authors’ information; (2) publication year; (3) demographic data including age, gender, diabetes duration, and background therapy; (4) baseline of outcome measures; (5) sample sizes; (6) interventions of each arm; (7) dosages of each arm; (8) outcomes of interest (see above); and (9) duration. For extension trials, data were extracted from the extension phase. Data that were not reported in the original manuscripts were retrieved from ClinicalTrials.gov (accessed on 22 August 2021). Two investigators (A.H.A. and A.I.F.) extracted data independently. The quality of eligible studies was evaluated according to the Cochrane Collaboration’s risk of bias tool for assessing risk of bias [26].

Data synthesis and analysis: The network meta-analysis was conducted based on the Cochrane institute instructions to compare the efficacy of weekly semaglutide 0.5 mg and 1 mg versus liraglutide 1.2 mg and 1.8 mg for the reduction of HbA1c and weight as a primary intervention [27]. Other medications went on the equation to complete the network of comparisons and they were; oral semaglutide 14 mg, liraglutide 3 mg, sitagliptin 100 mg, glimepiride 4 mg, glimepiride 8 mg, oral anti-diabetic drugs, exenatide 2 mg, and placebo. All continuous outcomes were performed using normal likelihood analysis and random effect was used for a better fit between trials. The NMA model was implemented using GeMTC software [28]. It used the Bayesian evidence network, which all indirect comparisons are taken into account to arrive at a single, integrated, estimate of the effect of all included treatments based on the included studies. The Bayesian analysis also allows assessing the consistency of the results to draw conclusions. Bayesian analysis is a type of analysis that is widely used to improve the estimate of the standard error used in a good old-fashioned *t*-test. The Bayesian has many advantages over the frequentist analysis; of them, it gives more coherent results that can be analyzed and interpreted in such a complicated review as network meta-analysis. Additionally, Bayesian inference allows for the flexible implementation of relatively complicated statistical techniques, such as those that involve hierarchical nonlinear model [29]. The results of the NMA was presented in mean treatment difference and 95% credible intervals (Crl) for mean treatment effect. The treatment that results in a greater treatment reduction from baseline was favored. Forest plots were generated using DistillerSR [30]. Surface under the cumulative ranking curve (SUCRA) was used for ranking interventions for each outcome [31].

## 3. Results

**Figure 1:**
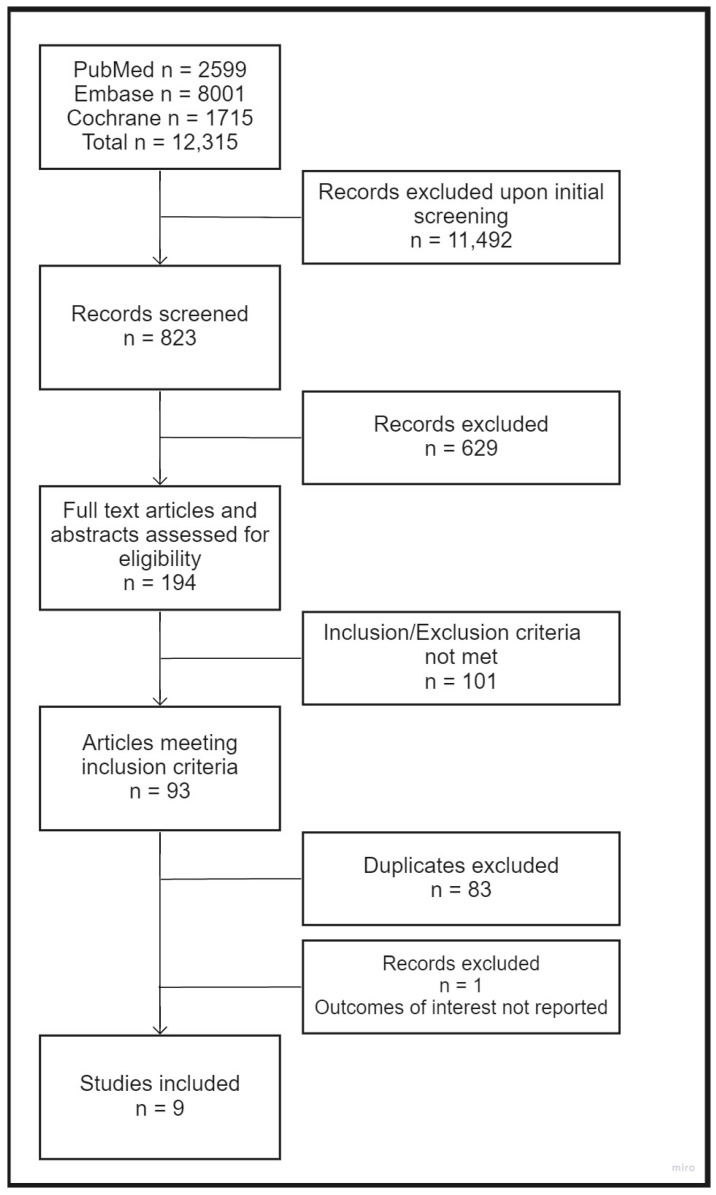
Flowchart of studies considered for inclusion.

The search process yielded 12,315 results. Of these, 11,492 results were manually excluded during initial screening, as we did not augment the search filter to exclude publications by language, animal vs. human design, published protocols, reviews, and so forth. By abstract screening, 629 results were excluded. This was followed by a full-text assessment. Of these, 101 were excluded as they did not meet the inclusion criteria. Furthermore, 85 results were excluded due to duplication or not reporting the outcomes of interest. As a result, nine studies were included in the final review. Flowchart of trial selection is shown in Figure 1.

### 3.1. Study Characteristics

Interventions included in the analysis once-weekly semaglutide 0.5 mg, once-weekly semaglutide 1 mg, once-daily liraglutide 1.2 mg, once-daily liraglutide 1.8 mg, once-daily oral semaglutide 14 mg, once-daily liraglutide 3 mg, once-weekly exenatide ER 2 mg, once-daily sitagliptin 100 mg, once-daily glimepiride 4 mg, once-daily glimepiride 8 mg, and metformin 1500–2000 mg daily. Overall, nine trials with a total of 9618 patients included to the analysis. The publication year ranged from 2009 to 2018. Trial duration ranged from 52 to 104 weeks. Trials design and baseline characteristics are shown in Table 1 and Table 2, respectively. Overall, the risk of bias was low in the included studies. However, due to limited long-term studies on liraglutide, 60% completion rate was accepted. Additionally, other biases that were due to the interference of the pharmaceutical companies in funding studies were permitted.

### 3.2. Network Meta-Analysis Results

**Figure 2:**
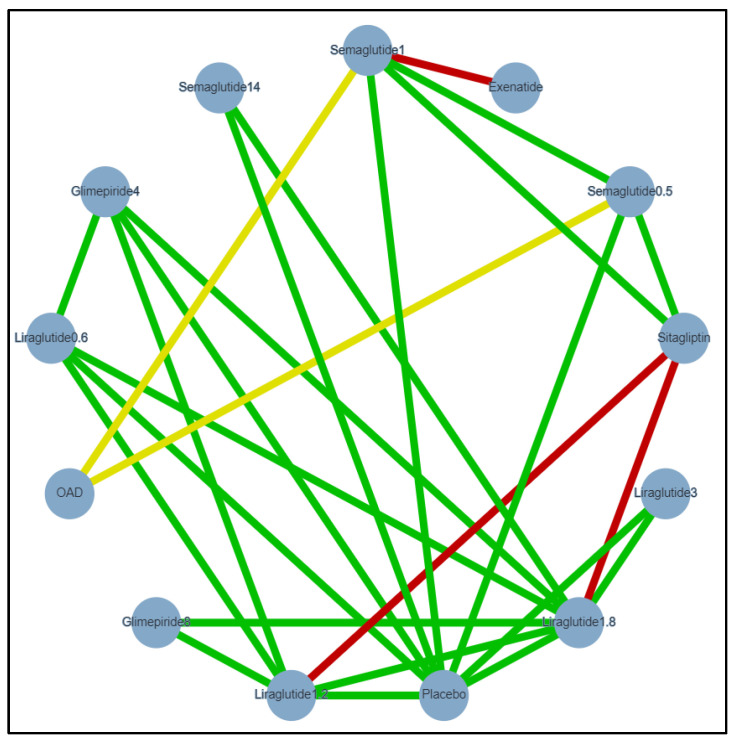
Evidence network of included studies for HbA1c analysis. Semaglutide 1 = Semaglutide 1 mg once-weekly (QW), Semaglutide 0.5 = Semaglutide 0.5 mg QW, Semaglutide 14 = oral semaglutide 14 mg, Glimpiride 4 = Glimepiride 4 mg, Glimpiride 8 = Glimepiride 8 mg, Liraglutide 0.6 = Liraglutide 0.6 mg once-daily (OD), Liraglutide 1.2 = Liraglutide 1.2 mg OD, Liraglutide 1.8 = Liraglutide 1.8 mg OD, Liraglutide 3 = Liraglutide 3 mg OD, OAD = Oral anti-diabetic drugs, Exenatide = Exenatide 2 mg, Sitagliptin = Sitagliptin 100 mg, HbA1c = glycated hemoglobin, Green: Low risk of bias, Yellow: Unclear risk of bias, Red: High risk of bias.

**Figure 3:**
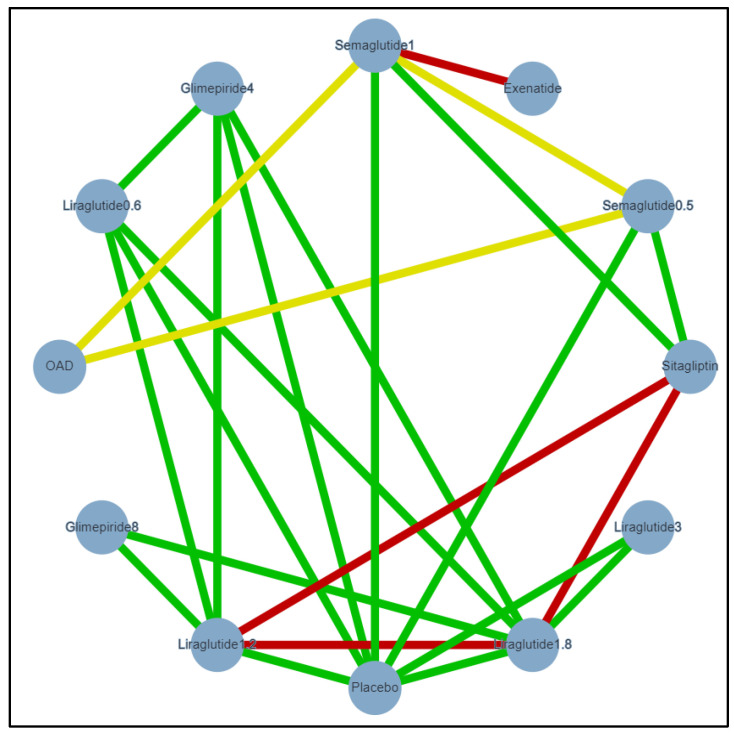
Evidence network of included studies for weight analysis. Semaglutide 1 = Semaglutide 1 mg once-weekly (QW), Semaglutide 0.5 = Semaglutide 0.5 mg QW, Glimpiride 4 = Glimepiride 4 mg, Glimpiride 8 = Glimepiride 8 mg, Liraglutide 0.6 = Liraglutide 0.6 mg once-daily (OD), Liraglutide 1.2 = Liraglutide 1.2 mg OD, Liraglutide 1.8 = Liraglutide 1.8 mg OD, Liraglutide 3 = Liraglutide 3 mg OD, OAD = Oral anti-diabetic drugs, Exenatide = Exenatide 2 mg, Sitagliptin = Sitagliptin 100 mg, Green: Low risk of bias, Yellow: Unclear risk of bias, Red: High risk of bias.

Two outcomes were analysed and presented in this NMA. A random-effect, meta-regression analysis was conducted on HbA1c and weight for a better model fit. Significant treatment differences were found as shown in the matrix, Table 3 and Table 4. The evidence network for Hba1c and weight analysis shown in Figure 2 and Figure 3, respectively.

**Figure 4:**
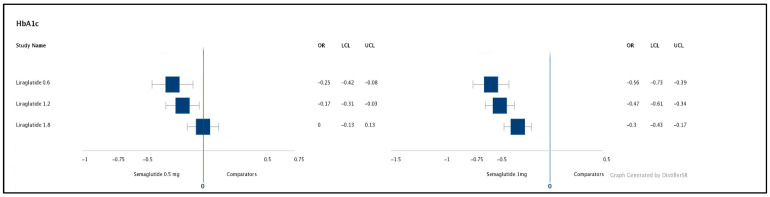
Forest plots of the NMA results: HbA1c outcomes for the primary comparators. The NMA results are presented as forest plots for a change from baseline in HbA1c. OR = treatment difference, LCL and UCL = lower and upper credible intervals (95% Crl) for mean treatment effect. Treatment differences are considered significant when the 95% CrI excludes 0. HbA1c = glycated hemoglobin, NMA = network meta-analysis.

HbA1c was reported in all nine studies. The HbA1c reduction from baseline was presented in the SUCRA score for ranking the main medications versus comparators, Table 5. Semaglutide 1 mg scored the highest in HbA1c reduction, 90.5% in SUCRA score, Table 5. As shown in the matrix, Table 3, semaglutide 1 mg QW found to be significantly superior to liraglutide 0.6 mg, 1.2 mg, 1.8 mg with a treatment difference of 0.56%, 0.47% and 0.3%, respectively. Semaglutide 0.5 mg QW was found to be significantly superior to liraglutide 0.6 mg and 1.2 mg with a treatment difference of 0.25% and 0.17%, respectively. The results of the NMA are presented as treatment differences in Figure 4.

**Figure 5:**
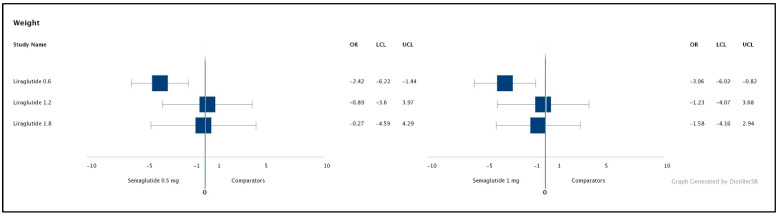
Forest plots of the NMA results: weight outcomes for primary comparators. The NMA results are presented as forest plots for a change from baseline in weight. OR = treatment difference, LCL and UCL = lower and upper credible intervals (95% Crl) for mean treatment effect. Treatment differences are considered significant when the 95% CrI excludes 0. NMA = network meta-analysis.

Eight out of nine studies reported weight reduction. The weight reduction from baseline was presented in the SUCRA score for ranking the main medications versus comparators, Table 5. Semaglutide 1 mg scored the highest in weight reduction, 84.9% in SUCRA score, Table 5. As shown in the matrix, Table 4, semaglutide 0.5 mg and 1 mg QW were significantly associated with greater weight reduction than liraglutide 0.6 mg with a treatment difference of 2.42 kg and 3.06 kg, respectively. However, no significant reduction was found in comparison to liraglutide 1.2 mg and 1.8 mg. The results of the NMA are presented as treatment differences in Figure 5.

## 4. Discussion

This network meta-analysis aimed to prove the long-term effects of semaglutide QW versus liraglutide OD on HbA1c and weight change in patients with T2DM. To our knowledge, no other NMA explored the long-term efficacy of semaglutide versus liraglutide. However, several reviews reported the early effects of semaglutide and liraglutide on HbA1c and weight change. 

Dose-dependent effect was seen in all the doses of interest, that is, semaglutide (0.5 mg, 1 mg) and liraglutide (1.2 mg, 1.8 mg). While, liraglutide 3 mg was included in a single RCT as part of this NMA, this might have led to the wide credible intervals and no significance.

In an NMA published in 2019 by Mishriky et al., semaglutide QW with other GLP-1 RA and dipeptidyl peptidase-4 inhibitors (DPP-4i) was compared in five trials with a duration of ≥12 weeks, where they found that semaglutide 1 mg was significantly superior in reducing HbA1c, with a change of −0.38% and −1.14%, respectively [40]. Additionally, a meta-analyses (MA) by Shi et al. focused on semaglutide RCTs found that semaglutide was more effective in glycemic control in comparison to exenatide and dulaglutide, with a significant difference of −0.47% [41]. However, they reported high heterogeneity (I^2^ = 92%) with regard to the study duration and dosage used [41]. Another MA published by Li et al. also compared semaglutide to placebo and other active comparators including exenatide and dulaglutide, where semaglutide showed further reduction of HbA1c with a change of 0.85% [42]. However, as disclosed in the Limitations section, the analysis may be restricted due to the significant heterogeneity (I^2^ = 94%), as the analysis included trials ranging from 12 weeks up to 104 weeks [42]. Furthermore, both MA used only direct comparison in their model. Thus, liraglutide was not included in the aforementioned MAs [41,42]. Our analysis suggested superiority on the long-term effects of semaglutide 1 mg QW over liraglutide OD, based on studies with a duration of ≥52 weeks, as shown by the SUCRA score in Table 5.

In an NMA published by Witkowski et al., semaglutide 1 mg QW was the most effective compared to other GLP-1 RA in reducing weight in 24 ± 4-week trials [21]. Again, our analysis showed constant superiority of semaglutide 1 mg QW over liraglutide OD even with longer-term use, as shown in the SUCRA score in Table 5. In a phase II trial by Nauck et al., comparing a 12-week weight change of semaglutide versus liraglutide, they reported statistically significant weight reduction with semaglutide versus liraglutide [43]. Both doses of once-weekly semaglutide 0.8 mg and 1.6 mg were greater in weight reduction compared to liraglutide 1.8 mg OD. However, doses of semaglutide used in the trial were experimental non-FDA approved. Additionally, the dose escalation protocol varied between the arms of semaglutide. The reported superiority of semaglutide 0.8 mg QW compared to liraglutide 1.8 mg was found to be consistent with the semaglutide 1 mg QW and semaglutide 0.5 mg QW generated in the SUCRA score of this NMA, Table 5. In the recently published SUSTAIN 10, a phase 3b trial, liraglutide 1.2 mg was compared against subcutaneous semaglutide 1 mg over a duration of 30 weeks. Semaglutide 1 mg showed significant superiority over liraglutide 1.2 mg with a treatment difference of 0.69% in HbA1c reduction. Due to the short duration of the trial, the presumed long-term effect of semaglutide was not reached as mentioned in the limitation section of the study [44]. Nevertheless, treatment differences reported in SUSTAIN 10 was supportive of this NMA results. An NMA by Webb et al. included long-term RCTs reported significant superiority of injectable semaglutide over other GLP-1 RAs including liraglutide. However, this NMA included only Japanese population using the Japanese protocol for liraglutide, a maximum dose of liraglutide 0.9 mg, which is not the recommended therapeutic dose by the manufacturing pharmaceutical company, and the U.S. FDA [14]. Additionally, the number of analyzed trials was considerably low, only four, with only a single trial including the therapeutic dose of semaglutide [45].

The strengths of this NMA includes the quality of the analyzed RCTs. In addition, to ensure all relevant RCTs were included, a through systematic literature review was conducted. Furthermore, the robustness of the results and conclusions were demonstrated across several sensitivity and restricted analyses. Exploratory meta-regression analyses also validated the choice model used for the key analyses, change in HbA1c and weight. Some common limitations were faced during this NMA, affecting the heterogeneity in the overall risk of bias. Analyses included different study designs, open-label, double-blind, and extension trials. In addition, there were variabilities in completion rates among the trials. Additionally, the low number of RCTs included in the NMA, a total of nine trials. Furthermore, some of the included RCTs were funded by the manufacturing pharmaceutical company. Moreover, this NMA included studies with multiple races and ethnicities while, no specific analysis was conducted regarding their distribution.

## 5. Conclusions

To date, no long-term phase III trials directly comparing semaglutide QW and liraglutide OD are available. Thus, the estimates driven from this NMA provide valuable evidence in the decision-making process for patients with T2DM. This NMA illustrated that semaglutide could improve the control of blood glucose and body weight. The capacity of long-term glycemic control and body weight reduction of injectable semaglutide appears to be more effective than other GLP-1 RAs, including liraglutide. However, considering the number of included studies and potential limitations, more large-scale, head-to-head, well-designed RCTs are needed to confirm these findings.

## Figures and Tables

**Table 1:** Design of phase III RCT in type 2 diabetes included in the NMA.

Study (Duration in Weeks)	Design	Treatment Arms	Inclusion Criteria	Primary Endpoint	Key Secondary Endpoints
LEAD-2 extension (104) [32]	Multinational, randomized, parallel assignment, open-label, active-comparator trial	Lira 0.6 mg, Lira 1.2 mg, Lira 1.8 mg, Glim 4 mg, PLA	18–80 years, diagnosed with T2DM, on OAD for >3 months, HbA1c 7–11%, BMI ≤ 40	Change in HbA1c	Change in weight
LEAD-3 (52) [33]	Multicenter, randomized, parallel assignment, double-blind, double-dummy, active-control trial	Lira 1.2 mg, Lira 1.8 mg, Glim 8 mg	18–80 years, diagnosed with T2DM, treated with diet/exercise or with not more than half maximal dose of OAD for >2 months, HbA1c 7–11%, BMI ≤ 45	Change in HbA1c	Change in weight
SUSTAIN-2 (56) [34]	Multinational, randomized, parallel assignment, double-blind, active-comparator trial	Sema QW 0.5 mg, Sema QW 1 mg, Sita 100 mg	≥18 years, diagnosed with T2DM, stable treatment OAD for >3 months (OAD: MET, PIO, ROSI, or combination), HbA1c 7–10.5%	Change in HbA1c	Change in weight
SUSTAIN-3 (56) [19]	Multinational, randomized, parallel assignment, open-label, active-comparator trial	Sema QW 1 mg, Exe 2 mg	≥18 years, diagnosed with T2DM, 1–2 OAD for >3 months (OAD: MET, TZD, or SU), HbA1c 7–10.5%	Change in HbA1c	Change in weight
SUSTAIN-6 (104) [35]	Multinational, randomized, parallel assignment, double-blind, placebo-controlled trial	Sema QW 0.5 mg, Sema QW 1 mg, 2 volume matched PLA	≥50 years, diagnosed with T2DM, antidiabetic drug naïve or on 1–2 OAD or insulin (NPH, long acting analogue, or premixed insulin), both types of insulin alone or with combination 1–2 OAD, HbA1c ≥ 7%	First occurrence of MACE *	Change in HbA1c, change in weight
SCALE (56) [36]	Multinational, randomized, parallel assignment, double-blind, placebo-controlled trial	Lira 3 mg, Lira 1.8 mg, PLA	≥18 years, diagnosed with T2DM, treated with diet/exercise or OAD (OAD: MET, TZD, or SU) or combination, HbA1c 7–10%, BMI ≥ 27	Change in weight	change in HbA1c
PIONEER-4 (52) [37]	Multinational, randomized, parallel assignment, double-blind, double-dummy, active-controlled and placebo-controlled trial	Sema OD 14 mg, Lira 1.8 mg, PLA	≥18 years, diagnosed with T2DM, treated with MET alone or in combination with SGLT-2 inhibitor >3 months, HbA1c 7–9.5%	Change in HbA1c	Change in weight
Pratley et al.; (52) [38]	Multinational, randomized, parallel group, open-label, active-comparator trial	Lira 1.2 mg, Lira 1.8 mg, Sita 100 mg	≥18 years, diagnosed with T2DM, treated with MET for >3 months, HbA1c 7.5–10%	Change in HbA1c	-
Kaku et al.; (56) [39]	Multicenter, single-country randomized, parallel group, open-label, active-controlled trial	Sema QW 0.5 mg, Sema QW 1 mg, OAD (one additional OAD + Pre-trial treatment)	≥20 years, diagnosed with T2DM, treated with diet/exercise for >1 month or OAD monotherapy (SU, Glinide, a-GI, TZD) for >2 months, HbA1c 7–10.5%	Emergent adverse events	Change in HbA1c, change in weight

Lira = Liraglutide, Sema QW = Semaglutide once weekly, Sema OD = Semaglutide once daily, Glim = Glimepiride, Sita = Sitagliptin, Exe = exenatide, OAD = Oral anti-diabetic drugs, PLA = Placebo, T2DM = Type 2 diabetes mellitus, HbA1C = Glycosylated hemoglobin, MET = Metformin, PIO = Pioglitazone, ROSI = Rosiglitazone, TZD = Thiazolidinedione, SU = Sulfonylurea, NPH = Neutral protamine Hagedorn, SGLT-2 = Sodium-glucose transport protein 2, a-GI = Alpha- glucosidase inhibitor, Glinide = Meglitinides, MACE = Major adverse cardiovascular events, N/R = Not reported.* MACE defined as cardiovascular death, non-fatal myocardial infarction, or non-fatal stroke.

**Table 2:** Baseline characteristics of included studies.

Study (Duration; Weeks)	Year	Arms	Sample Size	Age in Years Means (SD)	Male %	DOD Means (SD)	HbA1c Means (SD)	Weight Means (SD)	BMI Means (SD)
LEAD-2 extension (104)	2013	Lira 0.6 mg	880	56 (10.5)	62.4	7 (5)	8.4 (0.9)	88 (17)	N/R
		Lira 1.2 mg		57.2 (9.2)	53.8	7 (5)	8.3 (0.9)	88 (19)	N/R
		Lira 1.8 mg		56.8 (9.4)	58.7	8 (5)	8.3 (0.9)	88 (16)	N/R
		Glim 4 mg		57.3 (8.8)	57.4	8 (5)	8.4 (0.9)	89 (17)	N/R
		PLA		56 (9.4)	60	8 (6)	8.4 (1)	91 (17)	N/R
LEAD-3 (52)	2009	Lira 1.2 mg	746	53.7 (11)	46.6	5.2 (5.5)	8.3 (1)	92.5 (19.2)	33.2 (5.6)
		Lira 1.8 mg		52 (10.8)	49	5.3 (5.1)	8.3 (1.1)	92.8 (20.7)	32.8 (6.3)
		Glim 8 mg		53.4 (10.9)	53.6	5.6 (5.1)	8.4 (1.2)	93.4 (19.2)	33.2 (5.6)
SUSTAIN-2 (56)	2017	Sema QW 0.5 mg	1225	54.8 (10.2)	51	6.44 (4.7)	8 (0.92)	89.93 (20.39)	32.43 (6.2)
		Sema QW 1 mg		56 (9.4)	50	6.7 (5.6)	8 (0.93)	89.21 (20.74)	32.5 (6.6)
		Sita 100 mg		54.6 (10.4)	51	6.6 (5.9)	8.17 (0.92)	89.29 (19.67)	32.45 (5.8)
SUSTAIN-3 (56)	2018	Sema QW 1 mg	809	56.4 (10.3)	54.2	9 (6)	8.36 (0.95)	96.21 (22.5)	33.97 (7.2)
		Exe 2 mg		56.7 (11.1)	56.3	9.4 (6.7)	8.33 (0.96)	95.37 (20.46)	33.57 (6.2)
SUSTAIN-6 (104)	2016	Sema QW0.5 mg	3297	64.6 (7.3)	59.9	14.3 (8.2)	8.7 (1.39)	91.8 (20.25)	32.7 (6.29)
		Sema QW 1 mg		64.7 (7.1)	63	14.1 (8.2)	8.7 (1.51)	92.9 (20.05)	32.9 (6.18)
		PLA 1		64.8 (7.6)	58.5	14 (8.5)	8.7 (1.49)	91.8 (20.35)	32.9 (6.35)
		PLA 2		64.4 (7.5)	61.5	13.2 (7.4)	8.7 (1.45)	91.9 (20.75)	32.7 (5.97)
SCALE (56)	2015	Lira 3 mg	846	55 (10.8)	52	7.54 (5.65)	7.9 (0.8)	105.7 (21.9)	37.1 (6.5)
		Lira 1.8 mg		54.9 (10.7)	51.2	7.43 (5.16)	8 (0.8)	105.8 (21)	37 (6.9)
		PLA		54.7 (9.8)	45.8	6.71 (5.07)	7.9 (0.8)	106.5 (21.3)	37.4 (7.1)
PIONEER-4 (52)	2019	Sema OD 14 mg	711	56 (10)	52	7.8 (5.7)	8 (0.7)	92.9 (20.6)	32.5 (5.9)
		Lira 1.8 mg		56 (10)	52	7.3 (5.3)	8 (0.7)	95.5 (21.9)	33.4 (6.7)
		PLA		57 (10)	52	7.8 (5.5)	7.9 (0.7)	93.2 (20)	32.9 (6.1)
Pratley et al. (52)	2011	Lira 1.2 mg	497	55.9 (9.6)	51.6	6 (4.5)	8.4 (0.8)	N/R	32.6 (5.2)
		Lira 1.8 mg		55 (9.1)	52.5	6.4 (5.4)	8.4 (0.7)	N/R	33.1 (5.1)
		Sita 100 mg		55 (9)	54.8	6.3 (5.4)	8.5 (0.7)	N/R	32.6 (5.4)
Kaku et al. (56)	2018	Sema QW 0.5 mg	601	58 (10.6)	69.5	8.1 (6)	8 (0.9)	71 (15.4)	26.2 (4.8)
		Sema QW 1 mg		58.7 (10.2)	72.2	9.4 (6.5)	8.1 (1)	71.7 (15.9)	26.4 (4.7)
		OAD		59.2 (10.1)	74.2	9.3 (7)	8.1 (0.9)	72.2 (14.9)	26.7 (4.6)

Lira = Liraglutide, Sema QW = Semaglutide once weekly, Sema OD = Semaglutide once daily, Glim = Glimepiride, Sita = Sitagliptin, Exe = exenatide, OAD = Oral anti-diabetic drugs, PLA = Placebo, HbA1C = Glycosylated hemoglobin, N/R = Not reported, DOD = duration of diabetes, BMI = body mass index, SD = standard deviation.

**Table 3:** NMA matrix for HbA1c change from baseline: treatment difference.

	Exe 2 mg	Glim 4 mg	Glim 8 mg	Lira 0.6 mg	Lira 1.2 mg	Lira 1.8 mg	Lira 3 mg	OAD	PLA	Sema QW 0.5 mg	Sema QW 1 mg	Sema OD 14 mg
Exe 2 mg												
Glim 4 mg	−0.203 (0.428, 0.023)											
Glim 8 mg	0.250 (0.015, 0.484)	0.452 (0.272, 0.632)										
Lira 0.6 mg	−0.063 (−0.288, 0.163)	0.140 (−0.009, 0.289)	−0.312 (−0.492, −0.132)									
Lira 1.2 mg	−0.146 (−0.348, 0.057)	0.057 (−0.077, 0.191)	−0.395 (−0.531, −0.260)	−0.083 (−0.217, 0.051)								
Lira 1.8 mg	−0.315 (−0.512, −0.117)	−0.112 (−0.242, 0.018)	−0.565 (−0.700, −0.429)	−0.252 (−0.382, −0.122)	−0.169 (−0.253, −0.086)							
Lira 3 mg	−0.528 (−0.788, −0.269)	−0.326 (−0.544, −0.107)	−0.778 (−1.005, −0.551)	−0.466 (−0.684, −0.247)	−0.383 (−0.581, −0.185)	−0.214 (−0.398, −0.029)						
OAD	0.682 (0.480, 0.883)	0.884 (0.676, 1.093)	0.432 (0.213, 0.650)	0.744 (0.536, 0.953)	0.827 (0.643, 1.011)	0.997 (0.818, 1.175)	1.210 (0.965, 1.456)					
PLA	0.460 (0.270, 0.649)	0.662 (0.527, 0.797)	0.210 (0.055, 0.365)	0.522 (0.387, 0.657)	0.605 (0.502, 0.709)	0.775 (0.687, 0.862)	0.988 (0.804, 1.172)	−0.222 (−0.391, −0.053)				
Sema 0.5 mg	−0.317 (−0.488, −0.145)	−0.114 (−0.283, 0.055)	−0.566 (−0.748, −0.385)	−0.254 (−0.423, −0.085)	−0.171 (−0.309, −0.033)	−0.002 (−0.132, 0.128)	0.212 (−0.001, 0.425)	−0.998 (−1.135, −0.862)	−0.776 (−0.893, −0.659)			
Sema 1 mg	−0.620 (−0.769, −0.471)	−0.417 (−0.587, −0.248)	−0.870 (−1.051, −0.688)	−0.557 (−0.727, −0.388)	−0.474 (−0.612, −0.337)	−0.305 (−0.435, −0.175)	−0.092 (−0.304, 0.121)	−1.302 (−1.438, −1.165)	−1.080 (−1.197, −0.963)	−0.303 (−0.389, −0.218)		
Sema 14 mg	−0.578 (−0.806, −0.349)	−0.375 (−0.555, −0.195)	−0.828 (−1.017, −0.638)	−0.515 (−0.695, −0.335)	−0.432 (−0.586, −0.279)	−0.263 (−0.399, −0.127)	−0.049 (−0.270, 0.171)	−1.259 (−1.471, −1.048)	−1.037 (−1.174, −0.901)	−0.261 (−0.434, −0.088)	0.042 (−0.131, 0.216)	
Sita 100 mg	−0.369 (−0.764, 0.033)	0.571 (0.901, 0.242)	0.119 (−0.050, 0.288)	−0.431 (−0.780, 0.090)	−0.516 (−0.771, −0.263)	−0.684 (−0.931, 0.448)	−0.894 (−1.270, −0.517)	0.313 (−0.042, 0.684)	0.091 (−0.151, 0.349)	−0.684 (− 0.8, −0.57)	0.989 (0.871, 1.106)	−0.946 (−0.777, 1.115)

Lira = Liraglutide, Sema QW = Semaglutide once weekly, Sema OD = Semaglutide once daily, Glim = Glimepiride, Sita = Sitagliptin, Exe = exenatide, OAD = Oral anti-diabetic drugs, PLA = Placebo, HbA1C = Glycosylated hemoglobin, Not significant if crosses zero.

**Table 4:** NMA matrix for weight change from baseline: treatment difference.

	Exe 2 mg	Glim 4 mg	Glim 8 mg	Lira 0.6 mg	Lira 1.2 mg	Lira 1.8 mg	Lira 3 mg	OAD	PLA	Sema QW 0.5 mg	Sema QW 1 mg
Glim 4 mg	0.90 (−6.80, 8.72)										
Glim 8 mg	−1.85 (−10.17, 6.37)	−0.86 (−7.26, 5.32)									
Lira 0.6 mg	−1.74 (−4.64, 3.98)	0.81 (−2.35, 0.93)	0.86 (−0.37, 1.14)								
Lira 1.2 mg	−1.02 (−3.99, 1.95)	−1.08 (−2.77, 0.56)	−0.80 (−1.74, 0.51)	−1.31 (−5.85, 3.36)							
Lira 1.8 mg	−1.36 (−2.35, 1.47)	−2.45 (−4.03, 0.13)	−1.54 (−1.93, 1.11)	−1.63 (−6.23, 2.85)	−0.34 (−3.32, 2.58)						
Lira 3 mg	−1.66 (−14.68, 1.65)	−2.66 (−4.89, 0.78)	−2.73 (−4.55, 1.90)	−2.85 (−6.26, 3.37)	−1.56 (−7.13, 3.78)	−1.22 (−6.08, 3.57)					
OAD	0.89 (−1.04, 4.20)	1.93 (−5.20, 9.16)	1.09 (−6.44, 8.48)	−0.83 (−8.14, 6.41)	−2.09 (−8.55, 4.28)	2.45 (−3.76, 8.67)	−3.65 (−3.56, 11.11)				
PLA	0.31 (−4.30, 4.93)	1.32 (−3.32, 6.06)	0.48 (−5.06, 5.95)	1.45 (−3.20, 6.08)	2.72 (−1.09, 6.43)	3.09 (−0.20, 6.32)	4.30 (−0.57, 9.20)	0.64 (−6.42, 5.30)			
Sema 0.5 mg	−2.13 (−4.15, −1.89)	−4.19 (−8.16, −2.62)	1.97 (−4.34, 8.22)	−2.42 (−6.22, −1.44)	−0.89 (−3.60, 3.97)	−0.27 (−4.59, 4.29)	−0.48 (−3.51, 3.73)	−4.25 (−3.53, −6.13)	−3.84 (−2.09, −5.94)		
Sema 1 mg	−3.80 (−4.60, −2.96)	−4.03 (−4.97, −3.07)	−2.68 (−3.58, −1.77)	−3.06 (−6.02, −0.82,)	−1.23 (−4.07, 3.68)	−1.58 (−4.16, 2.94)	−0.82 (−3.36, 3.00)	−4.85 (−5.62, −3.88,)	−4.04 (−5.61, −2.47)	−0.35 (−3.59, 3.29)	
Sita 100 mg	1.06 (−1.58, 1.50)	1.09 (−1.47, 1.85)	0.19 (−2.55, 2.16)	1.32 (−4.24, 7.01)	0.01 (−4.13, 4.33)	0.31 (−3.61, 4.35)	1.51 (−4.39, 7.61)	−2.16 (−7.98, 3.86)	−2.77 (−6.98, 1.60)	0.25 (−2.38, 2.85)	0.90 (−3.19, 5.01)

Lira = Liraglutide, Sema QW = Semaglutide once weekly, Sema OD = Semaglutide once daily, Glim = Glimepiride, Sita = Sitagliptin, Exe = exenatide, OAD = Oral anti-diabetic drugs, PLA = Placebo, Not significant if crosses zero.

**Table 5:** Surface under the cumulative ranking curve (SUCRA) values for each intervention.

Treatment	HbA1c	Weight
Semaglutide 1 mg QW	0.9055	0.8486
Semaglutide 0.5 mg QW	0.7423	0.7308
Semaglutide 14 mg OD	0.7377	
Liraglutide 1.8 mg OD	0.7431	0.7193
Liraglutide 1.2 mg OD	0.7029	0.7165
Liraglutide 3 mg OD	0.5137	0.5340
Sitagliptin 100 mg	0.5039	0.4344
Liraglutide 0.6 mg OD	0.4514	0.4122
Glimepiride 4 mg	0.4083	0.4019
Glimepiride 8 mg	0.3237	0.3426
OAD	0.2198	0.3161
Exenatide 2 mg	0.2157	0.2085
Placebo	0.1085	0.0975

QW = once weekly, OD = once daily, OAD = Oral anti-diabetic drugs, HbA1C = Glycosylated hemoglobin. Green indicate highest score, blue is the second highest, and grey is not included.

## Data Availability

The datasets generated and/or analyzed during the current study are available from the corresponding author on reasonable request.

## Registration

This is a sub-study of the protocol registered in PROSPERO, number CRD42018091598.

## References

[1] Stumvoll M., Goldstein B.J., van Haeften T.W. (2005). Type 2 diabetes: Principles of pathogenesis and therapy. Lancet.

[2] Association A.D. (2016). Standards of Medical Care in Diabetes-2016: Summary of Revisions. Diabetes Care.

[3] Zoungas S., Chalmers J., Ninomiya T., Li Q., Cooper M.E., Colagiuri S., Fulcher G., De Galan B.E., Harrap S., Hamet P. (2012). Association of HbA1c levels with vascular complications and death in patients with type 2 diabetes: Evidence of glycaemic thresholds. Diabetologia.

[4] UK Prospective Diabetes Study (UKPDS) Group (1998). Effect of intensive blood-glucose control with metformin on complications in overweight patients with type 2 diabetes (UKPDS 34). Lancet.

[5] UK Prospective Diabetes Study (UKPDS) Group (1998). UKPDS 28: A randomized trial of efficacy of early addition of metformin in sulfonylurea-treated type 2 diabetes. Diabetes Care.

[6] Aroda V.R. (2018). A review of GLP-1 receptor agonists: Evolution and advancement, through the lens of randomised controlled trials. Diabetes Obes. Metab..

[7] Guja C., Miulescu R.D. (2017). Semaglutide—the “new kid on the block” in the field of glucagonlike peptide-1 receptor agonists?. Ann. Transl. Med..

[8] Chatterjee S., Davies M.J., Khunti K. (2018). What have we learnt from "real world" data, observational studies and meta-analyses. Diabetes Obes. Metab..

[9] Dhillon S. (2018). Semaglutide: First Global Approval. Drugs.

[10] Sharma A.K. (2018). Albiglutide: Is a better hope against diabetes mellitus?. Biomed. Pharmacother..

[11] (2017). Administration. 2016 Novel Drugs Summary. https://www.fda.gov/downloads/drugs/developmentapprovalprocess/druginnovation/ucm536693.pdf.

[12] (2017). Administration, Dulaglutide. https://www.accessdata.fda.gov/drugsatfda_docs/label/2017/125469s011s013lbl.pdf#page=21.

[13] Harris B.K., McCarty D.J. (2015). Efficacy and tolerability of glucagon-like peptide-1 receptor agonists in patients with type 2 diabetes mellitus. Adv. Endocrinol. Metab..

[14] Fda.gov. https://www.accessdata.fda.gov/drugsatfda_docs/label/2020/022341s036lbl.pdf.

[15] Trujillo M.J., Nuffer W., Ellis S.L. (2015). GLP-1 receptor agonists: A review of head-to-head clinical studies. Adv. Endocrinol. Metab..

[16] Madsbad S. (2016). Review of head-to-head comparisons of glucagon-like peptide-1 receptor agonists. Diabetes Obes. Metab..

[17] Sorli C., Harashima S.I., Tsoukas G.M., Unger J., Karsbøl J.D., Hansen T., Bain S.C. (2017). Efficacy and safety of once-weekly semaglutide monotherapy versus placebo in patients with type 2 diabetes (SUSTAIN 1): A double-blind, randomised, placebo-controlled, parallel-group, multinational, multicentre phase 3a trial. Lancet Diabetes Endocrinol..

[18] Pratley R.E., Aroda V.R., Lingvay I., Lüdemann J., Andreassen C., Navarria A., Viljoen A. (2018). Semaglutide versus dulaglutide once weekly in patients with type 2 diabetes (SUSTAIN 7): A randomised, open-label, phase 3b trial. Lancet Diabetes Endocrinol..

[19] Ahmann A.J., Capehorn M., Charpentier G., Dotta F., Henkel E., Lingvay I., Holst A.G., Annett M.P., Aroda V.R. (2018). Efficacy and Safety of Once-Weekly Semaglutide Versus Exenatide ER in Subjects With Type 2 Diabetes (SUSTAIN 3): A 56-Week, Open-Label, Randomized Clinical Trial. Diabetes Care.

[20] Courtney H., Nayar R., Rajeswaran C., Jandhyala R. (2017). Long-term management of type 2 diabetes with glucagon-like peptide-1 receptor agonists. Diabetes Metab. Syndr. Obes..

[21] Witkowski M., Wilkinson L., Webb N., Weids A., Glah D., Vrazic H. (2018). A Systematic Literature Review and Network Meta-Analysis Comparing Once-Weekly Semaglutide with Other GLP-1 Receptor Agonists in Patients with Type 2 Diabetes Previously Receiving 1-2 Oral Anti-Diabetic Drugs. Diabetes.

[22] Witkowski M., Wilkinson L., Webb N., Weids A., Glah D., Vrazic H. (2018). A Systematic Literature Review and Network Meta-Analysis Comparing Once-Weekly Semaglutide with Other GLP-1 Receptor Agonists in Patients with Type 2 Diabetes Previously Receiving Basal Insulin. Diabetes.

[23] Sutton A., Ades A.E., Cooper N., Abrams K. (2008). Use of indirect and mixed treatment comparisons for technology assessment. Pharmacoeconomics.

[24] Jansen J.P., Trikalinos T., Cappelleri J.C., Daw J., Andes S., Eldessouki R., Salanti G. (2014). Indirect treatment comparison/network meta-analysis study questionnaire to assess relevance and credibility to inform health care decision making: An ISPOR-AMCP-NPC Good Practice Task Force report. Value Health.

[25] Hutton B., Salanti G., Caldwell D.M., Chaimani A., Schmid C.H., Cameron C., Ioannidis J.P., Straus S., Thorlund K., Jansen J.P. (2015). The PRISMA extension statement for reporting of systematic reviews incorporating network meta-analyses of health care interventions: Checklist and explanations. Ann. Intern. Med..

[26] Higgins J.P., Altman D.G., Gøtzsche P.C., Jüni P., Moher D., Oxman A.D., Savović J., Schulz K.F., Weeks L., Sterne J.A. (2011). The Cochrane Collaboration’s tool for assessing risk of bias in randomised trials. BMJ.

[27] (2009). Cochrane Handbook for Systematic Reviews of Interventions Version 5.0.2 (Updated September 2009). http://www.cochrane-handbook.org.

[28] van Valkenhoef G. (2012). Automating network meta-analysis. Res. Synth. Methods.

[29] Wagenmakers E.J., Lodewyckx T., Iverson G.J., Hoijtink H., Klugkist I., Boelen P.A. (2008). Bayesian Versus Frequentist Inference. Bayesian Evaluation of Informative Hypotheses.

[30] DistillerSR Forest Plot Generator from Evidence Partners. https://www.evidencepartners.com/resources/forest-plot-generator/.

[31] Salanti G., Ades A.E., Ioannidis J.P. (2011). Graphical methods and numerical summaries for presenting results from multiple-treatment meta-analysis: An overview and tutorial. J. Clin. Epidemiol..

[32] Nauck M., Frid A., Hermansen K., Shah N.S., Tankova T., Mitha I.H., Zdravkovic M., Düring M., Matthews D.R. (2013). Long-term efficacy and safety comparison of liraglutide, glimepiride and placebo, all in combination with metformin in type 2 diabetes: 2-year results from the LEAD-2 study. Diabetes Obes. Metab..

[33] Garber A., Henry R., Ratner R., Garcia-Hernandez P.A., Rodriguez-Pattzi H., Olvera-Alvarez I., Hale P.M., Zdravkovic M., Bode B. (2009). Liraglutide versus glimepiride monotherapy for type 2 diabetes (LEAD-3 Mono): A randomised, 52-week, phase III, double-blind, parallel-treatment trial. Lancet.

[34] Ahrén B., Masmiquel L., Kumar H., Sargin M., Karsbøl J.D., Jacobsen S.H., Chow F. (2017). Efficacy and safety of once-weekly semaglutide versus once-daily sitagliptin as an add-on to metformin, thiazolidinediones, or both, in patients with type 2 diabetes (SUSTAIN 2): A 56-week, double-blind, phase 3a, randomised trial. Lancet Diabetes Endocrinol..

[35] Marso S.P., Bain S.C., Consoli A., Eliaschewitz F.G., Jódar E., Leiter L.A., Lingvay I., Rosenstock J., Seufert J., Warren M.L. (2016). Semaglutide and Cardiovascular Outcomes in Patients with Type 2 Diabetes. N. Engl. J. Med..

[36] Davies M.J., Bergenstal R., Bode B., Kushner R.F., Lewin A., Skjøth T.V., Andreasen A.H., Jensen C.B., DeFronzo R.A. (2015). Efficacy of Liraglutide for Weight Loss Among Patients With Type 2 Diabetes: The SCALE Diabetes Randomized Clinical Trial. JAMA.

[37] Pratley R. (2019). Oral semaglutide versus subcutaneous liraglutide and placebo in type 2 diabetes (PIONEER 4): A randomised, double-blind, phase 3a trial. Lancet.

[38] Pratley R. (2011). One year of liraglutide treatment offers sustained and more effective glycaemic control and weight reduction compared with sitagliptin, both in combination with metformin, in patients with type 2 diabetes: A randomised, parallel-group, open-label trial. Int. J. Clin. Pract..

[39] Kaku K. (2018). Safety and efficacy of once-weekly semaglutide vs additional oral antidiabetic drugs in Japanese people with inadequately controlled type 2 diabetes: A randomized trial. Diabetes Obes. Metab..

[40] Mishriky B.M., Cummings D.M., Powell J.R., Sewell K.A., Tanenberg R.J. (2019). Comparing once-weekly semaglutide to incretin-based therapies in patients with type 2 diabetes: A systematic review and meta-analysis. Diabetes Metab..

[41] Shi F.H., Li H., Cui M., Zhang Z.L., Gu Z.C., Liu X.Y. (2018). Efficacy and Safety of Once-Weekly Semaglutide for the Treatment of Type 2 Diabetes: A Systematic Review and Meta-Analysis of Randomized Controlled Trials. Front. Pharm..

[42] Li X., Qie S., Wang X., Zheng Y., Liu Y., Liu G. (2018). The safety and efficacy of once-weekly glucagon-like peptide-1 receptor agonist semaglutide in patients with type 2 diabetes mellitus: A systemic review and meta-analysis. Endocrine.

[43] Nauck M.A., Petrie J.R., Sesti G., Mannucci E., Courrèges J.P., Lindegaard M.L., Jensen C.B., Atkin S.L. (2016). A Phase 2, Randomized, Dose-Finding Study of the Novel Once-Weekly Human GLP-1 Analog, Semaglutide, Compared With Placebo and Open-Label Liraglutide in Patients With Type 2 Diabetes. Diabetes Care.

[44] Capehorn M.S., Catarig A.-M., Furberg J.K., Janez A., Price H.C., Tadayon S. (2020). Efficacy and safety of once-weekly semaglutide 1.0mg vs once-daily liraglutide 1.2mg as add-on to 1-3 oral antidiabetic drugs in subjects with type 2 diabetes (SUSTAIN 10). Diabetes Metab..

[45] Webb N., Orme M., Witkowski M., Nakanishi R., Langer J. (2018). A network meta-analysis comparing semaglutide once-weekly with other GLP-1 receptor agonists in Japanese patients with type 2 diabetes. Diabetes Ther..

